# Supplementary material for: Splice-Junction-Based Mapping of Alternative Isoforms in the Human Proteome
Source: Cell Rep. Author manuscript; Available in PMC 2020 Jan 15. (PMC6961840; doi:10.1016/j.celrep.2019.11.026)
Supplement: 2 [file NIHMS1546469-supplement-2.zip › DF1/PXD010154/Thyroid/Thyroid_13_TG_ERPQLWQTIQTQGHFQLQLPPGK.pdf]

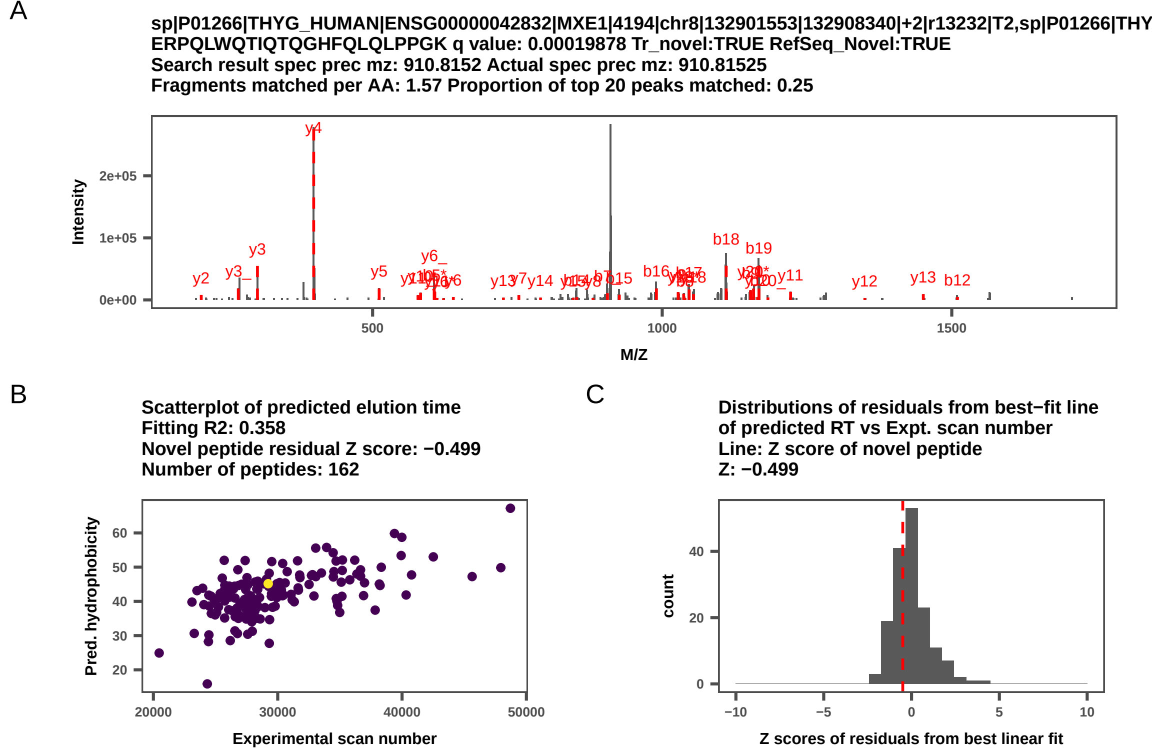
A
sp|P01266|THYG_HUMAN|ENSG00000042832|MXE1|4194|chr8|132901553|132908340|+2|r13232|T2,sp|P01266|THY
ERPQLWQTIQTQGHFQLQLPPGK q value: 0.00019878 Tr_novel:TRUE RefSeq_Novel:TRUE
Search result spec prec mz: 910.8152 Actual spec prec mz: 910.81525
Fragments matched per AA: 1.57 Proportion of top 20 peaks matched: 0.25
Intensity
2e+05
1e+05
0e+00
y4
y3
y2
y3_
y5
y6_
b18
b19
b16
y11
y12
y13
b12
y14
b15
500
1000
1500
M/Z
B
Scatterplot of predicted elution time
Fitting R2: 0.358
Novel peptide residual Z score: −0.499
Number of peptides: 162
Pred. hydrophobicity
60
50
40
30
20
20000
30000
40000
50000
Experimental scan number
C
Distributions of residuals from best−fit line
of predicted RT vs Expt. scan number
Line: Z score of novel peptide
Z: −0.499
count
40
20
0
−10
−5
0
5
10
Z scores of residuals from best linear fit
